# Tackling inequality and inequity in eye health: can the SDGs help us?

**Published:** 2016

**Authors:** Zoe Gray

**Affiliations:** Advocacy Manager: International Agency for the Prevention of Blindness (IAPB), London, UK. **zgray@iapb.org**

**Figure:**
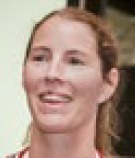
Zoe Gray

The Sustainable Development Goals (SDGs)^1^ were adopted at the United Nations (UN) General Assembly in September 2015. They are a set of goals and targets that all UN member states have committed to achieving: ‘to end poverty, protect the planet, and ensure prosperity for all’. A major emphasis of the SDGs is to ‘leave no one behind’; that is, to reach everyone, including the poor and the marginalised.

The health goal (Goal 3: Good health) is to ensure healthy lives and promote wellbeing for all, at all ages. One of the targets within Goal 3 is about universal health coverage (defined as access for all people to health services without suffering financial hardship). There is also considerable focus on attending to the needs of people with disabilities and vulnerable groups. Goal 3's emphasis on tackling health inequity and promoting access for all people complements the approach of the World Health Organization (WHO) action plan called Universal Eye Health: A Global Action Plan 2014–2019.^2^

## The importance for eye health

There are a number of targets and indicators within Goal 3: Good Health which are very relevant for eye health.

The inclusion of **Neglected Tropical Diseases (NTDs)** in the targets is a major achievement: the global indicator is the ‘number of people requiring interventions against NTDs’. If this is adequately addressed in national level indicators, policies and practices, it can significantly support efforts to manage and control blinding NTDs such as onchocerciasis and trachoma. These are largely diseases of poverty and addressing them can help to reduce eye health inequalities.

The indicator for the Goal 3 target on **health financing and human resources** includes a requirement for data collection on ophthalmologists at the national level. This information can help to strengthen advocacy to improving the size and distribution of the eye health workforce – which is essential to address rural/urban eye health inequalities.

The Goal 3 target about **universal health coverage** is very important. As countries progress on this, there should be opportunities to get eye health included within essential packages in social insurance or cost coverage schemes, which can greatly benefit eye health, including helping to reduce inequalities by reducing patients' out-of-pocket payments.

**Figure:**
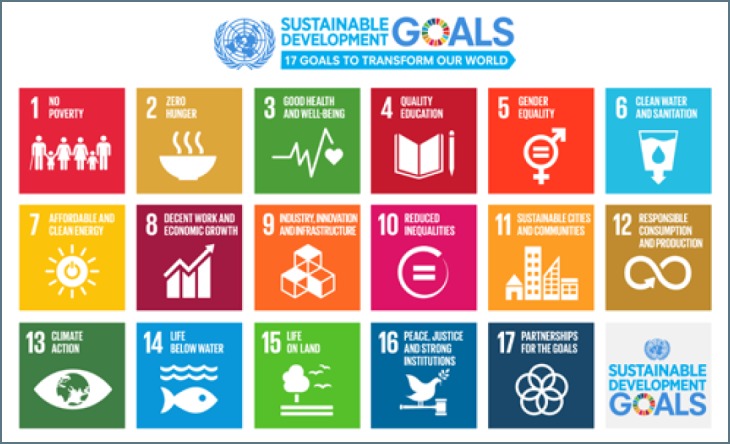


Currently, there is a significant political push for countries to mobilise their own resources, including financial resources, in order to meet their population's needs (e.g. health), rather than relying on international aid. This makes it critical to get involved with discussions about universal health coverage on national/country level.

There are other SDG goals and targets, such as on inclusive education, which may provide scope for advocating at the national level, such as promoting eye screening in schools as a means to improve access to inclusive education.

## Including eye health when implementing the SDGs

UN member countries have all made a major commitment to implement the SDGs. They are now starting to develop their own national action plan or strategy, with national targets and indicators to measure their progress against the SDG goals. National strategies and indicators will be particularly important as they will direct funding and government commitment towards programmes and services.

It is beneficial to get involved in these national processes and ensure that eye health targets and indicators are included – and that there is adequate action to achieve them. Here are a few examples.

- Ensuring that the national strategies, implementation and indicators adequately address the NTD indicator and target (in countries where blindness caused by NTDs remains a problem).
- Advocating for the inclusion of eye health services (for example trichiasis surgery, cataract surgery and low vision and rehabilitation services) within universal health coverage and social insurance schemes, in a way that enables access for the poorest and most marginalised.
- Lobbying to ensure that the health workforce indicator is taken up at national level and that there is a specific focus on eye health workers.
- Lobbying for the inclusion of cataract surgical coverage (CSC) as a national indicator, which will help prioritise action on cataract surgery. CSC is recognised within the WHO/World Bank universal eye health monitoring report^3^ as an important indicator of older people's access to health care, which can help to support arguments for CSC as an indicator.

The development of plans and indicators, and what part of government will take the lead, will differ from country to country. UN country teams, international agencies in-country and/or health ministries should be useful points of contact to advise about these processes. Working with other relevant organisations, both within and outside eye health, can be very effective, making it possible to deliver joint messages and lobby collectively.

## Accountability

Programmes and service delivery must be monitored to ensure that efforts are having the intended impact and reaching those most in need. Monitoring and research is needed to track progress and to support calls for new approaches when there are challenges or failures.

It is very likely that some countries will be selective and prioritise some of the SDG goals and targets rather than covering all of them. Advocacy is needed to hold governments to account and help them to achieve the stated ideals and aims of the SDGs.

There is an important role for the eye health community to promote the ideas of ‘leave no-one behind’ and equity, whether in service delivery or in advocacy.
